# Artemisinin Inhibits the Migration and Invasion in Uveal Melanoma via Inhibition of the PI3K/AKT/mTOR Signaling Pathway

**DOI:** 10.1155/2021/9911537

**Published:** 2021-12-11

**Authors:** Mohd Farhan, Marta Silva, Xing Xingan, Zhiwei Zhou, Wenhua Zheng

**Affiliations:** ^1^Cancer Center and Center of Reproduction, Development & Aging, Faculty of Health Sciences, University of Macau, Taipa, Macau SAR, China; ^2^Institute of Translation Medicine, Faculty of Health Sciences and Ministry of Education Frontiers Science Center for Precision Oncology, University of Macau, Taipa, Macau SAR, China

## Abstract

Uveal melanoma is the most common primary ocular neoplasm in adults, with many patients ending up developing liver metastasis and facing a significant reduction of their life expectancy due to the lack of efficient treatments. Artemisinin is an antimalarial drug that has been widely used in the clinic and whose anticancer properties have also been described. Its reported safety, affordability, and ability to reach the ocular tissues point that it has a potential therapeutic agent against uveal melanoma. In the present study, we found that a subantimalaria dosage of artemisinin significantly attenuated the migration and invasion potential of uveal melanoma cells, in a concentration-dependent manner. Assessment of the mechanisms underlying artemisinin anticancer action revealed that its use dramatically reduced the phosphorylation of PI3K, AKT, and mTOR in UM cells. Further inhibition of PI3K signaling, using LY294002, or of mTOR, by rapamycin, blocked the migration and invasion of UM cells similarly to artemisinin. In contrast, AKT or mTOR activator (Sc79 and MHY1485, respectively) attenuated the inhibitory effect of artemisinin on the migration and invasion abilities of UM cells, further validating that artemisinin's anticancer effect is likely to be mediated via inhibition of the PI3K/AKT/mTOR pathway. Artemisinin also induced mitochondrial membrane potential loss and apoptosis of UM cells, having no significant toxic effect on normal retinal neuronal cells RGC-5 and epithelial cells D407. These findings and the reported safety of artemisinin's clinical dosage strongly suggest the therapeutic potential of artemisinin in the prevention and treatment of uveal melanomas.

## 1. Introduction

Uveal melanoma (UM) is the most frequent primary intraocular malignancy among middle-aged people, affecting around four to seven patients per million individuals each year [1]. It originates from the melanocytes within the choroid, ciliary body, or iris and is usually asymptomatic [2]. The malignancy of UM correlates with its high propensity to metastasize, with 50% of all UM patients developing liver metastasis and facing a survival time between 4 and 15 months [3]. The reasons behind the aggressiveness of liver metastasis' progression are still unknown, and effective treatment is also not available. Therefore, a proper understanding of the pathophysiological mechanisms and related target molecular pathways is crucial to developing an effective therapeutic approach.

Drugs isolated from natural resources and natural drug analogues have been major contributors in the pharmacotherapy field for treating life-threatening diseases like cancer and infections [4, 5]. Artemisinin is a well-established drug used against malaria that is isolated from the sweet wormwood plant, also known as *Artemisia annua.* Besides its antimalarial effect, several studies suggest its potential against cancer, inflammation, viral infections, and other diseases [6]. In fact, artemisinin and its analogues have been reported to be able to successfully prevent different types of cancer, such as ovarian, colon, lung, breast, and pancreatic cancers [7–13]. Moreover, the concomitant administration of artesunate and standard chemotherapy was previously reported to extend the lifespan of two patients with uveal melanoma [14]. However, the mechanisms of action involved are still unclear, and more research is needed. Different putative mechanisms have been suggested to underlie the antitumorigenesis effect of artemisinin, including their supportive action on apoptosis and inhibitory effect on cell propagation and angiogenesis. Artesunate has also been described to play a key role in the apoptosis of human umbilical vein endothelial cells (HUVECs) via upregulation of BAX (Bcl2-associated X protein) and downregulation of Bcl2 (B-cell leukemia/lymphoma 2), a molecule that is reportedly overexpressed in UM [15, 16]. Artemisinins have also been described to suppress lung and colorectal cancer progression by inhibiting the Wnt/*β*-catenin pathway [17]. Importantly, artesunate was shown to block Wnt/*β*-catenin signaling and inhibit the growth, migration, and invasion of UM cells [18]. Studies performed in HUVECs revealed that artemisinin not only inhibited the migration, proliferation, and tube formation but also hindered the binding of vascular endothelial growth factor (VEGF) to VEGF receptor Flt-1 and KDR/flk-1 [19–21]. Artemisinins were also reported to be able to promote the downregulation of KDR/flk-1 in endothelial and tumor cells, as well as the decrease of human ovarian cancer growth, in an experimental animal model implanted with HO-8910 xenografts [20, 21]. Providing an adequate supply of oxygen and nutrients and more routes of dissemination to secondary sites, angiogenesis is fundamental for cancer progression and metastasis formation [22, 23]. The mitogen-activated protein kinase (MAPK) pathway has been found to play an important role in the development of different cancers, including UM [24]. Alterations of PI3K/AKT pathway signaling, an important regulator of cellular survival, have also been described. The downregulation of the tumor suppressor PTEN, a negative regulator of the PI3K/AKT cascade, suggests that the activation of this pathway constitutes a prosurvival strategy used by UM cells [25–27]. Curiously, artesunate was described to be able to inhibit PI3K/AKT signaling and to reduce allergic asthma [28] and TNF-*α*-induced production in human rheumatoid arthritis fibroblast-like synoviocytes [29, 30].

Since the therapeutic potential of artemisinin against UM metastatic potential is yet to be established, this study sought to assess its effect on the growth, migration, and invasion of UM cells and the related signaling pathways. Obtained results show that artemisinin impairs the proliferation, migration, and invasion abilities of UM cells via inhibition of the PI3K/AKT/mTOR pathway.

## 2. Materials and Methods

### 2.1. Materials

Uveal melanoma cells were acquired from Shanghai Bioleaf Biotech Co., Ltd. Human retinal pigment epithelial cell lines D407 and RGC-5 were obtained from the cell bank from Sun Yat-sen University (Guangzhou, China). Poly-L-lysine, DMSO (dimethyl sulfoxide), and bovine serum albumin were purchased from Sigma (St. Louis, MO, USA). DMEM, trypsin, fetal bovine serum (FBS), and penicillin-streptomycin (PS) were procured from Invitrogen (Carlsbad, USA). Hoechst 33342, 5,5′,6,6′-tetrachloro-1,1′,3,3′-tetraethyl-benzimidazolylcarbocyanineiodide (JC-1), and methylthiazolyltetrazolium (MTT) were obtained from Molecular Probes (Eugene, OR, USA). Sc79 (AKT activator) and MHY 1485 (mTOR activator) were purchased from Selleckchem Inhibitor Expert (Houston, TX, USA). LY294002 (PI3K inhibitor) and rapamycin (mTOR inhibitor) were obtained from Merck Millipore. Transwell invasion chambers were purchased from SPL Life Sciences (Korea), and matrix matrigel was obtained from BD Biosciences (San Jose, CA). Anti-*β*-actin, phospho-PI3K, total PI3K, phospho-AKT, total AKT, phospho-mTOR, total mTOR, and secondary antibody were purchased from Cell Signaling Technology (Woburn, USA). The Western blot kit was purchased from Bio-Rad Laboratories (USA). Crystal violet was from Sigma-Aldrich. 5% normal goat serum was from Vector Laboratories, Burlingame, CA. RIPA-lysis buffer (Millipore, Billerica, MA), PVDF membranes (Millipore), and LV-AKT1-RNAi (20908-2) were purchased by GeneChem.

### 2.2. Cell Culture

Uveal melanoma (UM-1), RGC-5, and D407 cells were cultured in Dulbecco's modified Eagle medium (DMEM) supplemented with 10% fetal bovine serum (FBS) and 1% penicillin/streptomycin solution. Cells were cultured in a T75cm^2^ flask and maintained in a humidified incubator at 37°C with 5% CO_2_. The culture medium was routinely changed every 2 days. UM-1 cells are the clone of uveal melanoma cancer cells isolated from tumor tissue of uveal melanin. RGC-5 is a neuronal precursor cell line, and D407 cells are human retinal cell lines that retain the pigments and epithelial characteristics after many passages.

### 2.3. Cellular Proliferation Assay

Cellular proliferation was analyzed using the MTT (3-(4,5-dimethylthiazol-2-yl)-2,5-diphenyltetrazolium) assay as described previously [31]. Briefly, the cells were seeded in 96-well plates at a density of 1 × 10^4^ cells/well. After overnight incubation, the cells were treated with 0, 3.125, 6.25, 12.5, 25, 50, and 100 *μ*M of artemisinin for 48 h. Six replicates were performed for each treatment. At the end of the incubation period, 10 *μ*L of MTT solution (5 mg/mL in PBS) was added to each well and incubated for 2 additional hours. After this time, the formazan crystals were dissolved in 150 *μ*L of DMSO, and the absorbance was recorded at 595 nm using Tecan Infinite® 200 PRO (Männedorf, Switzerland). Proliferation results are presented as a percentage of the control group.

### 2.4. Wound Healing Assay

The wound healing assay was performed to evaluate the effect of different concentrations of artemisinin on the migration ability of UM-1 cells as previously described [31]. Briefly, approximately 2 × 10^5^ cells were seeded in 6-well culture plates and cultured until 80-90% confluency. A wound was then created in the middle of the well using a sterile 200 *μ*L pipette tip, followed by rinsing with PBS to remove the cellular debris. This procedure was followed by the treatment of cells with 0, 3.125, 6.25, 12.5, 25, 50, and 100 *μ*M of artemisinin for 48 h. The photographs of wound closure were captured at 0, 24, and 48 h posttreatment using the ZOE Fluorescent Cell Imager (Hercules, California). The wound area was measured using ImageJ software version 1.47 h (http://imagej.nih.gov/ij). Cell migration results are presented as the percentage of the respective control groups.

### 2.5. Transwell Migration and Invasion Assays

To examine the effect of different concentrations of artemisinin on the invasive capacities of UM-1 cells, a matrigel-coated transwell invasion assay was performed following the manufacturer's protocol (BD Biosciences). Briefly, approximately 2 × 10^4^ cells were seeded on the upper well of matrigel-coated chambers (to assess transwell migration, the chambers were not coated with matrigel), placed in a 24-well plate with 450 *μ*L DMEM medium supplemented with 2% FBS containing 25 or 50 *μ*M of artemisinin. Chemoattractant (750 *μ*L DMEM medium supplemented with 20% FBS) was added to the lower wells, and the cells were incubated undisturbed in the CO_2_ incubator for 48 h. At the end of the incubation period, the cells located on the upper surface of the matrigel-coated or uncoated chambers were gently removed with the help of a cotton swab. The invasive/migrative cells, present on the lower surface of the membrane, were fixed using cold 100% methanol, stained with 0.1% crystal violet, and counted in at least 10 different microscopic fields. The results are expressed as the average number of invasive/migrative cells compared to the respective control.

### 2.6. Colony Formation Assay

UM cells (200 cells/well) were plated in 6-well plates and allowed to grow for 24 h. Then, the cells were treated with increasing concentrations of artemisinin from 3.125, 6.25, 12.5, 25, and 50 *μ*M. After 48 h of treatment with artemisinin, the culture medium was replaced with fresh medium, and the cells were allowed to grow freely for 10–14 days. After 14 days, the cells were washed with PBS three times and fixed with cold 4% paraformaldehyde. Cell colonies were stained with crystal violet (0.01% *w*/*v*), and the ones with more than 50 cells were counted using the ImageJ cell counter. All the experiments were carried out at least 3 times.

### 2.7. Apoptotic Nucleus Detection by Hoechst 33342 Staining

The Hoechst 33342 staining assay was performed to assess the effect of different concentrations of artemisinin on UM-1 cell apoptotic nuclei, as described previously [32]. Briefly, after treatment, UM-1 cells were fixed in 4% paraformaldehyde in 0.1 M phosphate buffer (PB) for 20 min. Nonspecific binding was blocked using 5% normal goat serum in 0.01 M PBS containing 0.3% Triton X-100 (PBST). The cells were washed twice with PBS and incubated with 5 *μ*g/mL Hoechst 33342 in PBS for 10 min at room temperature. The chromatin staining pattern was then analyzed for individual cells by fluorescence microscopy.

### 2.8. Flow Cytometry

Flow cytometry was used to assess cell apoptosis. Briefly, UM cells were trypsinized after the treatment, washed twice with 1x PBS, centrifuged at 1000 rpm for 5 min, and resuspended in Annexin V-FITC 1x binding buffer (195 *μ*L). The cells were supplemented with Annexin V-FITC (5 *μ*L) and incubated in the dark at 37°C for 20 min. After this period, the cells were centrifuged at 1000 rpm for 5 min, resuspended in 1x binding buffer (190 *μ*L), and incubated with propidium iodide (PI) (10 *μ*L) in the dark for 5 min. Apoptosis was quantified using the flow cytometer. Cell Quest Pro software (BD AccuriC6, BD, USA) was used for the analysis of the apoptosis condition.

### 2.9. JC-1 Mitochondrial Membrane Potential Assay

JC-1 dye was used to monitor mitochondrial membrane potential. Briefly, UM-1 cells were seeded into black bottom 96-well plates (1 × 10^4^ cells per well). After treatment with different doses of artemisinin, the cells were incubated with JC-1 (10 *μ*g/mL in medium) for 15 min at 37°C and then washed 3 times with PBS. For the red and green signal quantification, the intensity of red fluorescence (excitation 560 nm, emission 595 nm) and green fluorescence (excitation 485 nm, emission 535 nm) was analyzed by Infinite M200 PRO Multimode Microplate. Mitochondrial membrane potential (Δ*ψm*) was calculated as the ratio of JC-1 red/green fluorescence intensity normalized to the control group. Both fluorescent signals of cells were also measured and recorded by fluorescent microscopy.

### 2.10. Western Blot Analysis

UM cells were seeded in 60 mm dishes and treated with 0, 3.125, 6.25, 12.5, 25, 50, and 100 *μ*M of artemisinin, for 24 h. Whole cell lysates were prepared using RIPA-lysis buffer following the manufacturer's instructions. A total of 40 *μ*g of protein was resolved on 10-12% SDS-PAGE and transferred onto PVDF membranes, followed by incubation with blocking buffer (5% skimmed milk or 1% BSA in TBST) for 1 h. Thereafter, the membranes were incubated with the primary antibodies specific for p-PI3K, PI3K, p-Akt, Akt, p-mTOR, mTOR, and *β*-actin overnight at 4°C. After primary antibody incubation, the membranes were washed in TBST and incubated with the appropriate secondary antibodies conjugated with horseradish peroxidase (HRP) for 1 h at room temperature. Protein bands were visualized using the enhanced chemiluminescence detection substrate (Millipore) on an ImageQuant LAS4010 chemidocumentation system (GE Healthcare, Amersham, UK). Proteins' expression was quantified using ImageJ software version 1.47 h (http://http://imagej.nih.gov/ij) and normalized with the respective *β*-actin bands.

### 2.11. Subcutaneous UM Tumor Mouse Model

The establishment of an UM subcutaneous tumor mouse model was performed as previously described with slight modifications [33], using two-month-old nude mice of 25-gram average body weight. UM cells were harvested during the exponential growth phase, transferred to suspension using 0.1% Trypsin, washed thrice with ice-cold PBS, and then resuspended in PBS. Nude mice were then injected subcutaneously with UM cell suspension (1 × 10^6^ cells/100 *μ*L) on one of the sides of their posterior flank. After injection, the mice were randomly divided into three experimental groups (six animals per group): control, 40 mg/kg artemisinin (1 dose/day), and 40 mg/kg artemisinin (2 doses/day with five-hour interval between doses). Artemisinin was administered by intraperitoneal (i.p.) injection for 25 days. Mouse weight and tumor sizes were evaluated every other day. At the end of the experiment, all the mice were sacrificed, and all the tumor tissues were collected, photographed, and preserved for further investigation. All animal experiments were performed following the standard protocol approved by the Animal Research Ethics Committee (AREC) of the Faculty of Health Sciences, University of Macau (protocol No. UMARE-015-2017).

### 2.12. Statistical Analysis

Statistical analysis was performed using GraphPad Prism 5.0 statistical software (GraphPad software, Inc., San Diego, CA, USA). All experiments were performed in triplicate. Data are expressed as the mean ± standard deviation (SD). Statistical analysis was carried out using one-way ANOVA, followed by Tukey's multiple comparisons, with *p* < 0.05 considered statistically significant.

## 3. Results

### 3.1. Artemisinin Attenuated the Migration, Invasion, and Colony Formation Ability of UM Cells

**Figure 1:**
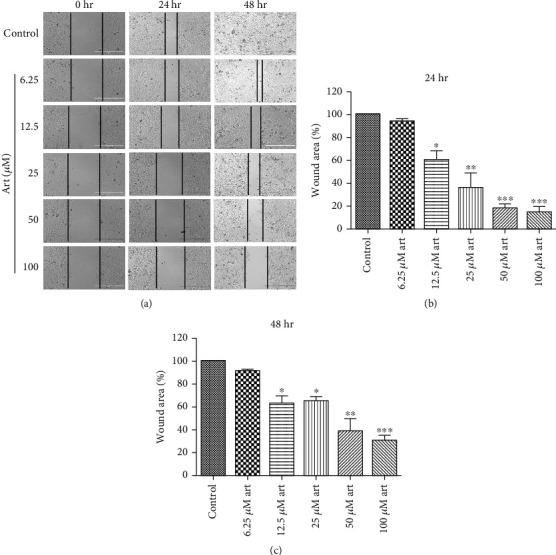
Artemisinin significantly inhibited the migration of UM cells in a dose-dependent manner. (a) After creating a wound in the middle of the well, UM cells were treated with increasing concentrations of artemisinin (6.25-100 *μ*M); (b, c) cell migratory capacity was assessed at 24 and 48 h by calculating the wound closure area. The results represent the mean ± SD of three independent experiments. ^∗^*p* < 0.05,  ^∗∗^*p* < 0.01, and^∗∗∗^*p* < 0.001 were considered statistically significant.

**Figure 2:**
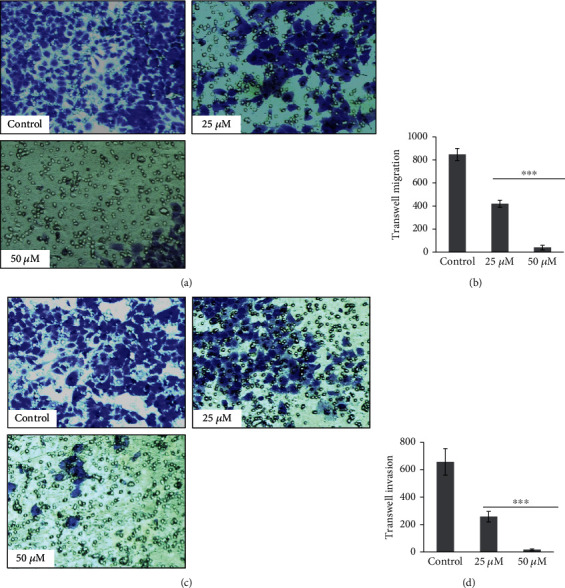
Artemisinin significantly inhibited the transwell migration and invasion ability of UM cells in a dose-dependent manner. UM cells were seeded in transwell inserts with or without coating of matrigel and treated with artemisinin for 48 hours. (a, b) Transwell migration and number of migrated UM cells; (c, d) transwell invasion and number of invasive UM cells. The results represent the mean ± SD of three independent experiments versus the control group. ^∗^*p* < 0.05,  ^∗∗^*p* < 0.01, and^∗∗∗^*p* < 0.001 were considered statistically significant.

**Figure 3:**
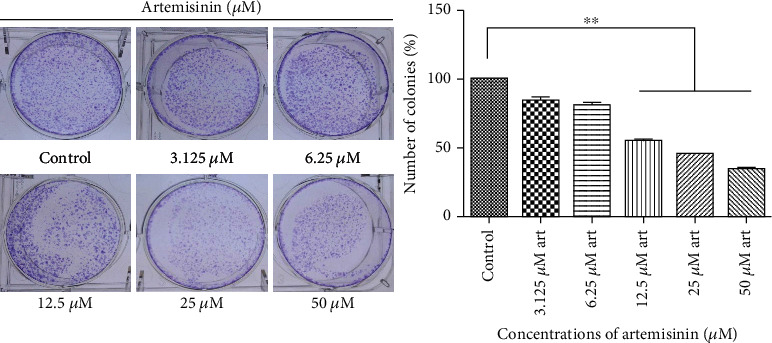
Artemisinin attenuates the colony formation ability of UM cells. Cells were treated with different concentrations of artemisinin for 10-15 days, and the colonies with more than 50 cells were counted. Control was set as 100%, and data was calculated as a percentage of the corresponding control. All the results represent the mean ± SD of three independent experiments versus the control group. ^∗∗^*p* < 0.01 was considered statistically significant.

Artemisinin treatment had an inhibitory effect on the migration and invasion abilities of UM cells that was dose-dependent (Figures 1 and 2). The wound healing assay allowed evaluation of the effect of different concentrations of artemisinin on the cells' migration and proliferation. Obtained results revealed that after 24 hours, artemisinin significantly prevented the reduction of the wound healing area in a concentration-dependent manner, starting at 12.5 *μ*M (Figures 1(a) and 1(b)). After 48 hours, untreated cells were able to achieve complete wound closure, which was avoided in the wells with cells treated with artemisinin (Figures 1(a) and 1(c)). These results were further confirmed by the transwell migration assay, whose results showed that artemisinin significantly decreased the number of migrative cells (Figures 2(a) and 2(b)). Consistent with these results, artemisinin treatment also significantly decreased the number of invasive cells attached to the bottom of the transwell chamber (Figures 2(c) and 2(d)). Artemisinin treatment also suppressed the colony formation ability of UM cells (Figure 3).

### 3.2. Artemisinin Promoted Cellular Apoptosis and Mitochondrial Membrane Potential Loss

**Figure 4:**
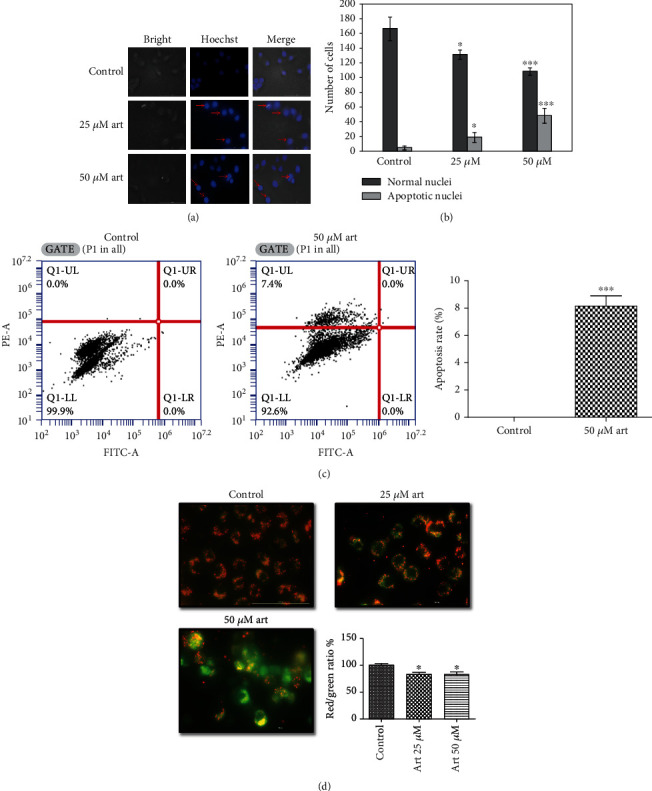
Artemisinin promoted cellular apoptosis and the loss of mitochondrial membrane potential. (a, b) Apoptotic nuclei were detected by Hoechst 33342 staining assay and counted. (c) Apoptosis was determined by flow cytometry. (d) Mitochondrial membrane potential was determined by JC-1 dye (10 *μ*g/mL in medium). Artemisinin treatment promoted the decrease of red to green fluorescence intensity ratio, indicative of mitochondrial membrane potential loss. All the results represent the mean ± SD of three independent experiments versus the control group. ^∗^*p* < 0.05,  ^∗∗^*p* < 0.01, and^∗∗∗^*p* < 0.001 were considered statistically significant.

**Figure 5:**
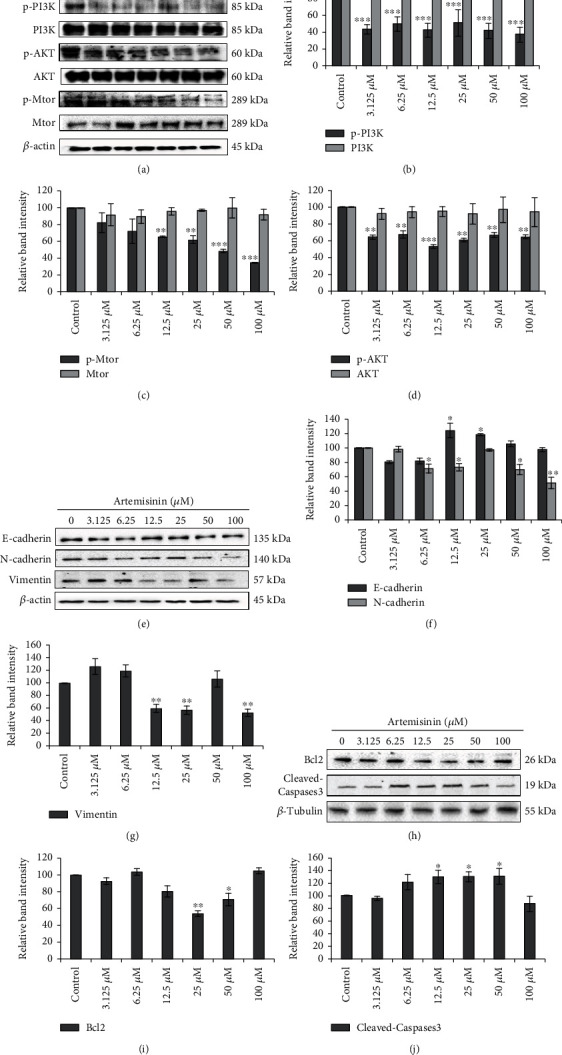
Artemisinin inhibited PI3K/AKT/mTOR signaling pathway in UM cells. (a, e, h) UM cells were treated with different doses of artemisinin, and Western blot analysis was performed to detect p-PI3K, PI3K, p-AKT, AKT, p-mTOR, mTOR, E-cadherin, N-cadherin, Vimentin, Bcl2, and cleaved caspase 3 expression. (e) Artemisinin altered the expression levels of EMT markers in UM cells. (h) Artemisinin decreased the protein expression of Bcl2 and increased the protein expression of cleaved caspase 3, suggesting an increase in apoptosis. *β*-Actin and *β*-tubulin were used as loading controls. (b–d, f, g, i, j) Quantification of the bands in (a). All the results represent the mean ± SD of three independent experiments versus the control group. ^∗^*p* < 0.05,  ^∗∗^*p* < 0.01, and^∗∗∗^*p* < 0.001 were considered statistically significant.

Hoechst staining was used to investigate the effect of artemisinin treatment on cellular apoptosis. Incubation of UM cells with 25 *μ*M or 50 *μ*M of artemisinin promoted an increased number of apoptotic and condensed nuclei in a dose-dependent manner (Figures 4(a) and 4(b)). Consistent with these results, flow cytometry analysis also showed an increase in apoptosis in cells treated with artemisinin (Figure 4(c)). The mitochondria are highly involved in the process of apoptosis induction, and cancer cells usually display hyperpolarized mitochondrial membranes in comparison with healthy cells. Artemisinin treatment promoted the decrease of mitochondrial membrane potential denoted by the decreased red/green fluorescence intensity ratio (Figure 4(d)). Western blot analysis of Bcl2 and cleaved caspase 3 protein levels also revalidated our results showing the increase of cellular apoptosis by the decreased expression of Bcl2 and increased levels of cleaved caspase 3 after treatment of artemisinin in UM cells (Figure 5(h)).

### 3.3. Artemisinin Inhibited PI3K/AKT/mTOR Pathway in UM Cells

**Figure 6:**
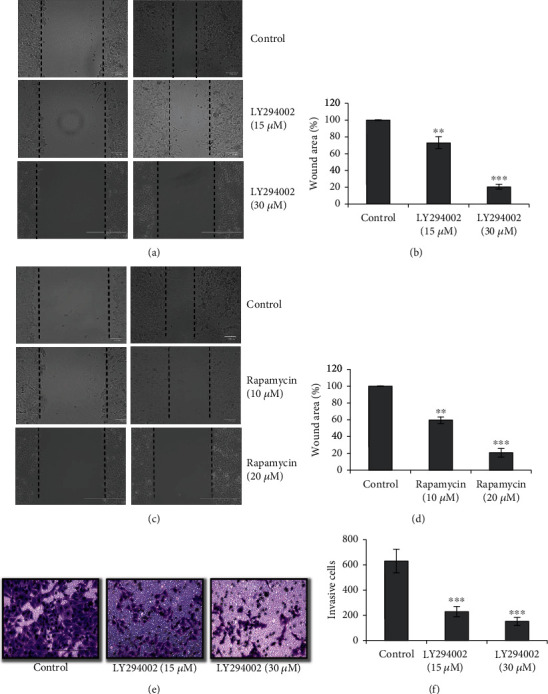
Treatment with LY294002 (PI3K inhibitor) or rapamycin (mTOR inhibitor) decreased UM cell migration, and LY294002 treatment also inhibited the invasion in a dose-dependent manner. (a, c) Effect of LY294002 or rapamycin on the migration of UM cells after 24 h incubation in a wound healing assay. (b, d) Quantification of the wound closure area. (e) Treatment with LY294002 also decreased the invasion of UM cells. (f) Quantification of invasive cells. All the results represent the mean ± SD of three independent experiments versus the control group. ^∗^*p* < 0.05,  ^∗∗^*p* < 0.01, and^∗∗∗^*p* < 0.001 were considered statistically significant.

To evaluate the molecular pathways involved in the effect of artemisinin on UM cell migration ability, the cells were incubated with different doses of artemisinin and the activation of PI3K, AKT, and mTOR was determined by Western blotting. Obtained results showed that artemisinin significantly inhibited the phosphorylation of PI3K, AKT, and mTOR in a dose-dependent manner (Figure 5(a)). This is indicative that the inhibitory effect of artemisinin on UM cell migration is mediated, at least in part, by PI3K/AKT/mTOR signaling pathway inhibition. Consistent with this, treatment of cells with LY294002 (PI3K inhibitor) and rapamycin (mTOR inhibitor) promoted the decrease of UM cell migration ability in a dose-dependent manner (Figure 6). LY294002 also inhibited the invasion of UM cells in a dose-dependent manner (Figures 6(e) and 6(f)). E- and N-cadherins are EMT markers that play a key role in the epithelial-mesenchymal process, and in our study, artemisinin also promoted the increase and decrease of E-cadherin and N-cadherin expression, respectively. Artemisinin treatment also resulted in the decrease of Vimentin expression levels further suggesting its involvement in the protein levels of EMT markers and supporting the antimetastatic role of artemisinin in UM cells (Figure 5(e)).

### 3.4. Activation of AKT and mTOR Signaling Suppressed the Effect of Artemisinin Treatment on UM Cells

**Figure 7:**
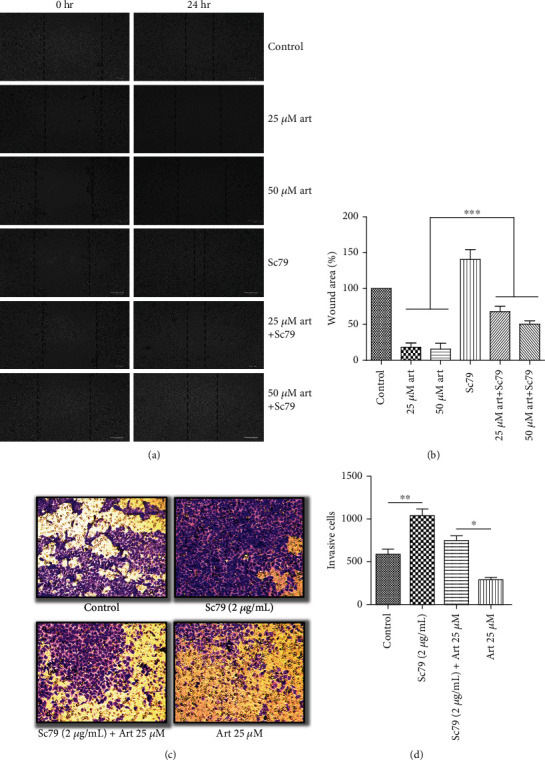
Treatment of cells with an AKT activator (Sc79) attenuated the inhibitory effect of artemisinin on cell migration and invasion. The cells were pretreated with Sc79 followed by incubation with different concentrations of artemisinin. (a, b) Treatment with Sc79 prevented the decrease of the wound closure area promoted by artemisinin. (c, d) Sc79 increased the number of artemisinin-treated invasive cells. All the results represent the mean ± SD of three independent experiments versus the control group. ^∗^*p* < 0.05, ^∗∗^*p* < 0.01, and ^∗∗∗^*p* < 0.001 were considered statistically significant.

**Figure 8:**
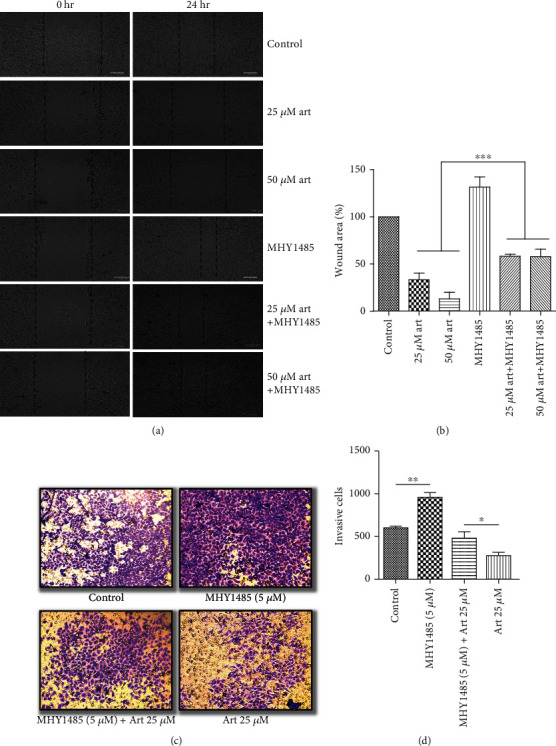
Treatment of cells with mTOR activator (MHY1485) attenuated the inhibitory effect of artemisinin on cell migration and invasion. The cells were pretreated with MHY1485 followed by incubation with different concentrations of artemisinin. (a, b) Treatment with MHY1485 prevented the decrease of the wound closure area promoted by artemisinin. (c, d) MHY1485 increased the number of artemisinin-treated invasive cells. All the results represent the mean ± SD of three independent experiments versus the control group. ^∗^*p* < 0.05,  ^∗∗^*p* < 0.01, and^∗∗∗^*p* < 0.001 were considered statistically significant.

Further validation of the mechanistic pathways involved in the effect of artemisinin treatment was performed by incubating the cells with Sc79 (AKT activator) (Figure 7) or MHY1485 (mTOR activator) (Figure 8), before treatment with different doses of artemisinin. Obtained results revealed that the activation of the AKT/mTOR pathway suppressed the inhibitory effect of artemisinin on cellular migration and invasion, further validating the involvement of the PI3K/AKT/mTOR signaling pathway on artemisinin action.

### 3.5. Silencing of AKT1 by RNAi Inhibits the Migration and Invasion and Enhances the Artemisinin Antimigratory and Anti-invasive Effect on UM Cells

**Figure 9:**
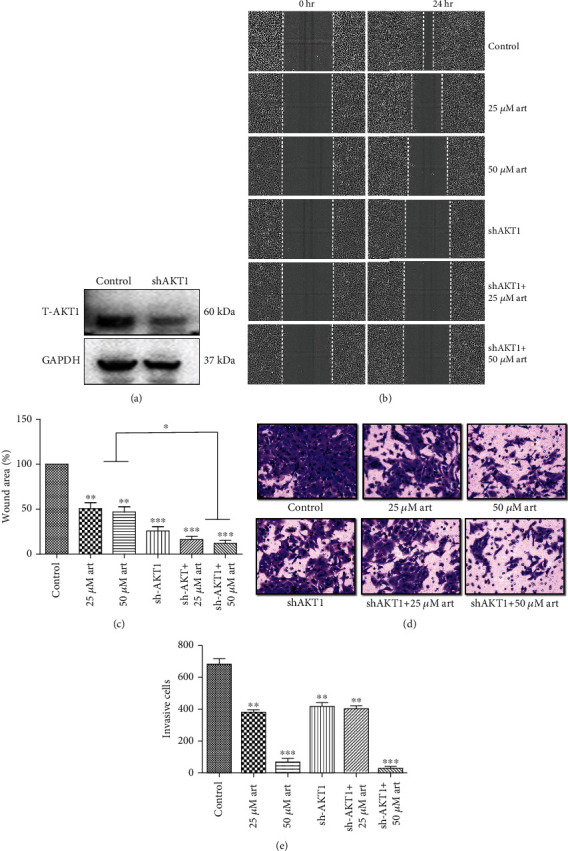
Silencing of AKT1 by RNAi inhibited the migration and invasion and enhanced artemisinin antimigratory and anti-invasive effect on UM cells. UM cells were treated with RNAi (AKT1 silencing) to form AKT1 silenced stable cells. These stable cells were used for further migration and invasion study assays with different doses of artemisinin. AKT1 silencing inhibited the migration and invasion ability of UM cells and also enhanced the antimigratory and anti-invasive effect of artemisinin. All the results represent the mean ± SD of three independent experiments versus the control group. ^∗^*p* < 0.05,  ^∗∗^*p* < 0.01, and^∗∗∗^*p* < 0.001 were considered statistically significant.

The involvement of AKT in the effect of artemisinin treatment was assessed by incubating the cells with RNAi (AKT1 silencing) prior to artemisinin treatment and followed by the performance of the migration and invasion assays. Obtained results showed that AKT1 silencing not only inhibited the cells' migration and invasion abilities but also enhanced artemisinin antimigratory and anti-invasive effect (Figure 9). These results further validate the involvement of the PI3K/AKT/mTOR signaling pathway in artemisinin action.

### 3.6. Artemisinin Inhibited Tumor Growth in a Mouse Model of Subcutaneous Uveal Melanoma

**Figure 10:**
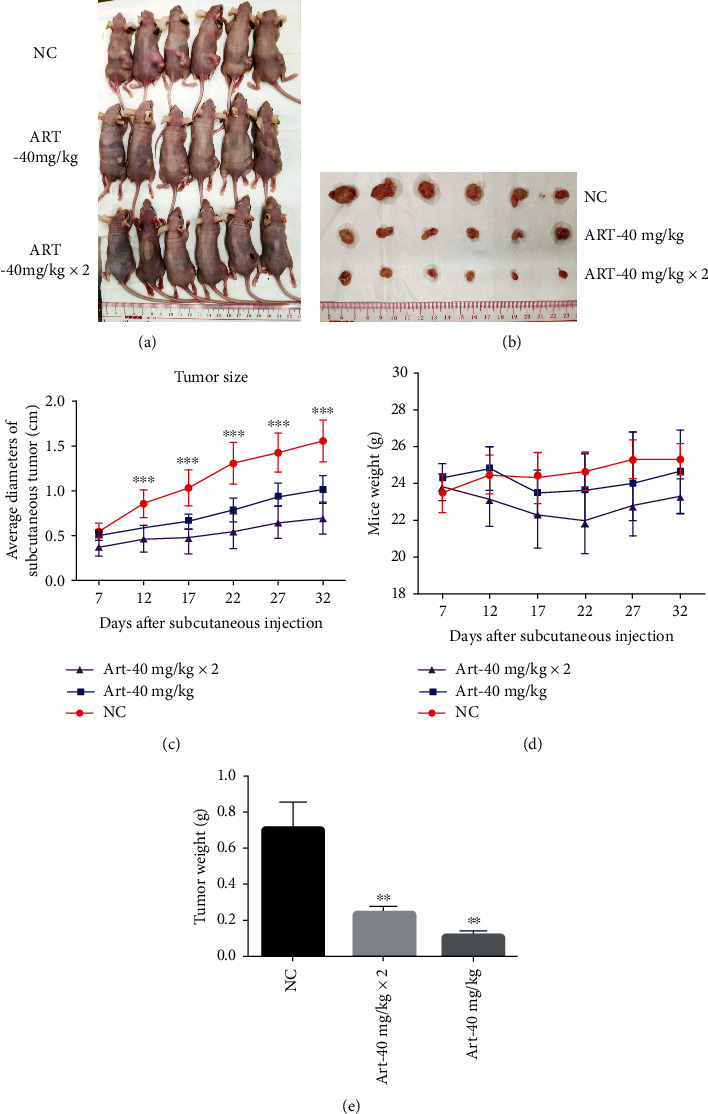
Artemisinin inhibited tumor growth in a mouse model of subcutaneous uveal melanoma. (a) Images of the tumors before isolation. (b) Average diameter of the tumors throughout the experiment. (c) Images of the isolated tumors and their size. (d) Tumor weight after isolation. (e) Weight of animals throughout the experiment. All the results represent the mean ± SD. ^∗^*p* < 0.05 and^∗∗^*p* < 0.01 versus control.

The mouse model of subcutaneous uveal melanoma has been extensively used for the assessment of the therapeutic action of cytotoxic chemicals. Upon subcutaneous injection, UM cells form a tangible tumor within five to seven days. Therefore, we sought to use this model to investigate *in vivo* the antitumor effect of artemisinin. Obtained results showed that the body weight of the mice in all experimental groups receiving artemisinin was not affected (Figure 10(d)). At the end of the experiment, all tumors were collected (Figure 10(b)), and their volume was calculated (Figure 10(c)), indicating that artemisinin significantly reduced the growth of UM melanoma tumors by almost 50% in the animals treated with a lower dose (40 mg/kg (1 dose/day)) and 75% in the animals from the higher dose group (40 mg/kg (2 doses/day)). Artemisinin significantly reduced the uveal melanoma tumor size in treated mice. These results demonstrate that artemisinin inhibited the tumor growth of uveal melanoma *in vivo* in a dose-dependent manner.

### 3.7. Artemisinin Showed No Toxicity in Normal Ocular Cell Lines

**Figure 11:**
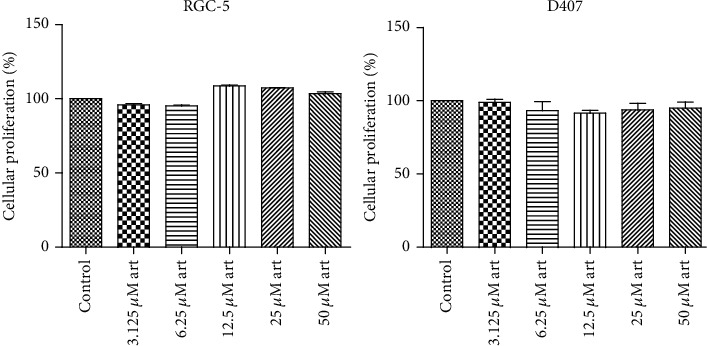
RGC-5 and D407 cells were treated with the indicated concentrations of artemisinin for 48 h, and cell viability was evaluated by the MTT assay. All the results represent the mean ± SD of three independent experiments versus the control group. ^∗^*p* < 0.05, ^∗∗^*p* < 0.01, and ^∗∗∗^*p* < 0.001 were considered statistically significant.

Assessment of a possible toxic effect of artemisinin treatment in normal ocular cell lines revealed that the range of doses tested had no toxicity as demonstrated by the cell viability results (Figure 11).

## 4. Discussion

This study describes the effect of artemisinin against the proliferation, migration, and invasion of UM cells via inhibition of the PI3K/AKT/mTOR pathway, suggesting its potential use in therapeutic strategies targeting UM.

Approximately 50% of all patients diagnosed with uveal melanoma are at risk of developing liver metastases and consequently facing a dramatic shortage of their life expectancy. This process of metastasis formation and preferential dissemination to the liver is complex, and the underlying mechanisms are still unclear. Therefore, the search for potential compounds able to decrease the proliferation, migration, and invasion of UM cells constitutes an important step for the establishment of novel effective therapeutic strategies against UM.

Artemisinin is a well-established antimalarial drug, whose potential use in other complications aside malaria has been widely explored [34]. Artemisinin therapeutic effect on different cancers has also been previously described, and the results from this study contribute to the body of knowledge of the antitumorigenesis effect of these antimalarial drugs. By describing for the first time that a subclinical antimalarial dose of artemisinin inhibited the migration, invasion, and cellular proliferation of UM cells, these findings suggest the potential of artemisinin-based therapies for UM metastasis prevention or treatment. Artemisinin also attenuated the ability of UM cells to form colonies, an important feature of cancer progression. Further study revealed that artemisinin promoted the loss of mitochondrial membrane potential and the apoptosis of UM cells, while having no significant toxic effect on RGC-5 and D407 cells, suggesting its specific action towards the malignant phenotype. E-cadherin, N-cadherin, and Vimentin are key players in the epithelial-mesenchymal process that is involved in cancer metastasis. This study shows that artemisinin is able to affect the expression levels of these markers, supporting its antimetastatic action in UM cells. Further study of artemisinin action *in vivo* showed that it was able to inhibit tumor growth in an UM subcutaneous mouse model. Artemisinin effect was shown to be dose-dependent as it significantly reduced the growth of UM melanoma tumors by almost 50% in the animals treated with the lower dose and 75% in the animals receiving a higher dose. The body weights of the animals receiving artemisinin were not affected further suggesting that it was able to affect tumor growth without having a toxic effect.

Previous studies performed in human breast cancer cells revealed that trisubstituted imidazole drugs targeting the oncogenic PI3K/AKT/mTOR pathway can lead to apoptosis of breast cancer cells [35]. Alterations of the PI3K/AKT pathway in UM cells have been previously reported with studies suggesting that the activation of this pathway contributes to the growth and survival of UM cells and that increased levels of AKT phosphorylation are linked with a poor prognosis in different UMs [25–27, 36]. Moreover, hepatocyte growth factor- (HGF-) induced migration of UM cells was shown to be dependent on PI3K/AKT signaling, whose activation promotes the attenuation of cell-cell adhesion and enhances their motility and migration abilities [26]. Despite the well-known importance of this pathway in tumor cell growth and proliferation, the first drug able to specifically inhibit PI3K was only approved recently for breast cancer treatment and a second one is currently undergoing clinical trials (http://ClinicalTrial.gov ID: NCT04191499) [37]. Many of the efforts towards the inhibition of PI3K/AKT signaling involve its indirect inhibition by targeting mTOR, a major downstream target of PI3K that has also been reported to be activated in UM cells [38]. mTOR plays a crucial regulatory role in cell metabolism and growth. Previous reports suggest that the PI3K/AKT/mTORC1 signaling pathway is highly activated in almost 70% of ovarian cancers and tumor tissue of gastric cancers [39]. In this study, artemisinin treatment inhibited PI3K/AKT/mTOR signaling suggesting that its antitumorigenic effect may occur via targeting of this pathway. Specific inhibition of PI3K or mTOR (by LY294002 or rapamycin, respectively) had a negative impact on the cell migration ability that was dose-dependent. These findings are in accordance with previous studies reporting an inhibitory effect on UM cell proliferation, viability, and tumor growth upon the inhibition of PI3K and mTOR [38, 40]. Contrarily to our results, the antitumor effect of mTOR inhibition has been described to be attenuated in UM due to the possible occurrence of a feedback mechanism that may promote AKT activation [38]. In addition, in some UM cell lines, mTOR activation seems to be independent of AKT signaling [40, 41]. In our study, activation of AKT (Sc79) or mTOR (MHY1485) enhanced the migration and invasion abilities of UM cells and attenuated the inhibitory effect of artemisinin, further suggesting its involvement in the mediation of artemisinin's anticancer effect.

**Figure 12:**
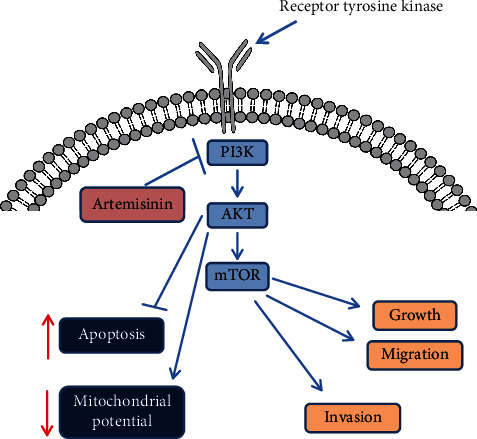
Possible mechanism involved in the anticancer effect of artemisinin.

In conclusion (Figure 12), our results suggest the potential use of artemisinin in the prevention of UM progression. By acting in PI3K/AKT/mTOR, a pathway with recognized importance in cancer, artemisinin was shown to be able to impair the migration, invasion, and proliferation of UM cells.

## Data Availability

The data used to support the findings of this study are included within the article.
